# Involvement of Abscisic Acid in PSII Photodamage and D1 Protein Turnover for Light-Induced Premature Senescence of Rice Flag Leaves

**DOI:** 10.1371/journal.pone.0161203

**Published:** 2016-08-17

**Authors:** Fubiao Wang, Jianchao Liu, Minxue Chen, Lujian Zhou, Zhaowei Li, Qian Zhao, Gang Pan, Syed-Hassan-Raza Zaidi, Fangmin Cheng

**Affiliations:** 1 Institute of Crop Science, College of Agriculture and Biotechnology, Zhejiang University, Hangzhou 310058, China; 2 State Key Laboratory of Rice Biology, China National Rice Research Institute, Hangzhou 310006, China; 3 Jiangsu Collaborative Innovation Center for Modern Crop Production, Nanjing, China; University of Hyderabad, INDIA

## Abstract

D1 protein in the PSII reaction center is the major target of photodamage, and it exhibits the highest turnover rate among all the thylakoid proteins. In this paper, rice *psf* (premature senescence of flag leaves) mutant and its wild type were used to investigate the genotype-dependent alteration in PSII photo-damage and D1 protein turnover during leaf senescence and its relation to ABA accumulation in senescent leaves. The symptom and extent of leaf senescence of the *psf* mutant appeared to be sunlight-dependent under natural field condition. The *psf* also displayed significantly higher levels of ABA accumulation in senescent leaves than the wild type. However, the premature senescence lesion of *psf* leaves could be alleviated by shaded treatment, concomitantly with the strikingly suppressed ABA level in the shaded areas of flag leaves. The change in ABA concentration contributed to the regulation of shade-delayed leaf senescence. The participation of ABA in the timing of senescence initiation and in the subsequent rate of leaf senescence was closely associated with PSII photodamage and D1 protein turnover during leaf senescence, in which the transcriptional expression of several key genes (*psbA*, *psbB*, *psbC* and *OsFtsH2*) involved in D1 protein biosynthesis and PSII repair cycle was seriously suppressed by the significantly increased ABA level. This response resulted in the low rate of D1 protein synthesis and impaired repair recovery in the presence of ABA. The *psf* showed evidently decreased D1 protein amount in the senescent leaves. Both the inhibition of de novo synthesized D1 protein and the slow rate of proteolytic removal for the photodamaged D1 protein was among the most crucial steps for the linkage between light-dependent leaf senescence and the varying ABA concentration in *psf* mutant leaves. *OsFtsH2* transcriptional expression possibly played an important role in the regulation of D1 protein turnover and PSII repair cycle in relation to ABA mediated leaf senescence.

## Introduction

Leaf senescence is a key developmental step in plant life cycle. During this process, leaf cells undergo complex metabolic changes and sequential degeneration in the cellular structure. These changes include several hallmarks, such as the visible color yellowing in leaf phenotype, which is due to a significant loss of chlorophyll (Chl), decline in leaf photosynthesis potential, destruction of chloroplast structures, and the translocation of nutrients to other parts of the plant, thus eventually leading to the death of the senescing leaf [1–3]. Leaf senescence syndrome occurs in an age-dependent manner, but the initiation and progression of leaf senescence can be largely regulated by various environmental and endogenous factors [4–6]. Many studies have suggested that leaf senescence is an active, highly organized form of programmed cell death (PCD). Senescence activates a self-destructive program to degenerate cellular structures and enables a leaf to make its final contribution to the plant by remobilizing the nutrients accumulated in the senescing leaf [1,3,7]. In plant leaves, the chloroplasts are the major cellular organelles in a photosynthetic cell, and up to 80% of total leaf nitrogen is reserved in the chloroplast [8]. Chloroplast breakdown is a central theme and is particularly important in leaf senescence process, not only because chloroplasts represent the single most important source of remobilizing nutrients in a leaf, but also because their degradation causes a decline in the photosynthetic activity and Chl content of leaves [9,10]. More recent studies have shown that the degradation of the integral structure of protein membranes and hydrophilic membrane-associated proteins in senescent leaves are typically initiated inside chloroplasts, and subsequently proceed with a vacuolar organelle catalyzed by proteinases [8,11,12]. The chloroplasts are dismantled before deteriorative changes can be detected in other organelles [13–15]. Therefore, it is a crucially important to elucidate the mechanism and regulation of chloroplast protein degradation during senescence for crop yield improvement.

Photosystem II (PSII) in chloroplast is known as a pigment-protein complex that is embedded in the chloroplast thylakoid membrane and is related to an electronic carrier for light absorption, transmission and transformation. PSII is highly vulnerable to photo-oxidative damage and requires a sophisticated quality control system called PSII repair cycle [9,13,16]. The functional PSII complex comprises one of two reaction center proteins (D1 and D2), the Chl *a* binding core antennae proteins (CP43 and CP47), light-harvesting chlorophyll complex II (LHCII), a Mn cluster (Mn 4) and a plastquinone pool among others. The D1 and D2 subunits form a heterodimer, Chl *a* molecular bound by CP43 and CP47 act to relay excitation energy from the peripheral light-harvesting complex to the reaction center [17,18]. Many studies have revealed that excess light energy absorbed by photosynthetic pigments is responsible for PSII photodamage [1,7,19], in which D1 protein in PSII reaction centre is the major target of photodamage and exhibits the highest turnover rate among all the thylakoid proteins [16,17]. When higher plants are exposed to excessive light, the reaction center-binding protein D1 of the PSII is damaged by the reactive oxygen species produced near PSII, thus leading to a decrease in PSII activity [1]. D1 protein is encoded by the *psbA* gene, and the photo-inactivated PSII complex may be repaired rapidly by D1 protein synthesis [20]. D1 turnover in PSII repair cycle involves the proteolytic removal of the damaged D1 protein by FtsH proteases located in the chloroplast, followed by the coordinated insertion of newly synthesized D1 protein into the thylakoid membrane [21,22]. Under various types of environment stress, the rate of PSII repair cycle and D1 protein turnover is impaired because of the inhibition of the de novo synthesized D1 protein and the acceleration of the damaged D1 protein [18,23,24]. Previous studies also revealed that light accelerated D1 protein degradation, whereas the darkening treatment significantly delayed the D1 protein damage [17,23,25]. However, how the initiation and progression of plant leaf senescence is implemented by PSII photo-oxidative damage and D1 protein turnover in the chloroplast thylakoid membrane under light exposure remains to be clarified, although leaf senescence can be easily characterized by the loss of chlorophyll (Chl) and the destruction of the chloroplast membrane structure.

The plant hormone abscisic acid (ABA) is an important phytohormone that regulates many developmental processes and the adaptive responses of plants to various stresses including drought, salinity, low temperature, and other biotic factors [26,27]. Extensive evidence has revealed that ABA is involved in various physiological events and signal transduction pathways that regulated numerous genes that are expressed at specific growth stages or as a result of environmental stress [28–30]. Under stressful conditions, ABA accumulation in plant leaves induce the generation of ROS, concomitantly with the increasing malondialdehyde (MDA) content in plant leaves [30–31]. Interestingly, the enhancement of ABA induced by water deficiency may promote the translocation of remobilizing carbon from senescing leaves to grain organs, in addition to ABA-induced stomatal closure [32,33]. Furthermore, the accumulation of ABA in plant leaves affect the rate of photosynthetic electron transport system, with the increasing level of endogenous ABA in senescing leaves [32,33]. Exogenous ABA application has been confirmed to promote leaf senescence in a wide range of plant species [1,26,27], and a decrease in delayed fluorescence and protein contents is detected to be coincident with ABA-induced leaf senescence [34,35]. Thus, ABA has been widely considered as one of the most effective plant hormones in terms of promoting leaf senescence [27,35]. However, there are few reports about the interplay between light-accelerated D1 protein degradation and ABA accumulation in rice leaves, and our understanding of the underlying mechanism of PSII photodamaged and D1 protein turnover in relation to ABA induced leaf senescence remains poor.

In this study, we compared the genotypic differences in the temporal patterns of Chl content, PSII fluorescence parameters, ABA accumulation, and D1 protein degradation during leaf senescence using the early leaf senescence mutant rice (*psf*) and its wild type. Interestingly, no visible difference in plant phenotype and leaf senescence was observed between the *psf* mutant and the wild type at seedling and tillering stages. Moreover, the Chl content and the photosynthetic rate of flag leaves in *psf7954* were measured to be virtually similar to those in its wild type on the pre-anthesis day. However, the flag leaves of *psf* mutant exhibited the senescence symptom after anthesis, and the extent of exacerbated senescence in the phenotype appeared to be sunlight-dependent under field growth conditions. Thus, this mutant is an ideal material for investigating whether or not the initiation and progression of sunlight-dependent senescence was regulated by PSII photodamage and D1 protein turnover/degradation in senescing leaves, as well as their relationship with ABA accumulation during leaf senescence. Furthermore, the triggering effect of exogenous ABA concentration on the transcriptional expression of various genes related to D1 protein turnover/degradation is further investigated to clarify the effect of altering the ABA level on D1 protein turnover/degradation for ABA-mediated leaf senescence.

## Materials and Methods

### Plant materials and experiment treatments

Two rice genotypes, an excellent *indica* restorer line (Zhehui7954) and its corresponding mutant with the premature senescence of flag leaves (*psf*) occurred after anthesis, were used in the study. The *psf* mutant was derived from Zhehui7954 cultivar (*Oryza sativa* L. ssp. *indica*) mature seeds with gamma irradiation as mutant factor, and the stably inherited mutant was obtained through the successive self-pollination for more than eight generations. Field experiments were performed in 2013–2015 at the experimental station of Zijingang campus (30°18′N, 120°04′E) Zhejiang University in Hangzhou, China. Germinating rice seeds were sown on April 21 and transplanted on May 20. The field plots performed following a random design in field. Triplicates with three plots were arranged for each genotype, and each plot was planted in 10×12 rows with plant spacing of 17 cm×22 cm. Field management was performed on the basis of local practices, and the paddy soil type was subjected to periodical water-logging, with a total N of 1.70 g/kg, available P of 24.6 mg/kg and exchangeable K of 104.1 mg/kg. The sampling was carried out during grain filling stage. 60–70 panicles with uniform anthesis day were randomly selected and tagged at the full heading day. The flag leaves were sampled from the full heading day to maturity at a 7-day interval (0 day to 28 days after anthesis). All samples were quickly immersed in liquid nitrogen and kept at -80°C until further analysis.

According to previous preliminary observations, the symptom and extent of leaf senescence of *psf* mutant appeared to be sunlight-dependent under the natural field growth condition. To investigate the impact of sunlight on the initiation and progression of leaf senescence and its underlying relationship with D1 degradation and ABA accumulation in the senescing leaves, we conducted an experiment of different shaded treatments for the flag leaves of rice plants grown under field natural condition, with a continuous sunshine weather being chosen in September 2013 on the basis of local forecast. The shaded treatments were implemented as follows: Treatment 1: The flag leaves of *psf* mutant were shaded with a piece of 2–3 cm aluminum foil before the emergence of its senescence lesions. After 7 d shaded treatment, the shaded and un-shaded areas of rice leaves were sampled for further analysis after camera photograph; Treatment 2: The flag leaves of *psf* mutant were shaded for 7 d with a piece of 2–3 cm aluminum foil after the emergence of lesions (6^th^ day after anthesis), the shaded and un-shaded areas of full leaves were separately sampled for assaying D1 protein and ABA concentration; Treatment 3: The aluminum foil shading on the treated leaves (Treatment 2) was removed after 7 d shaded treatment, and then the shaded area was re-exposed to sunlight. The re-exposed and un-shaded areas of full leaves were separately sampled for further analysis after 7 d-term solar irradiation. Lesion development was documented by digital camera.

The detached flag leaves of *psf* and its wild type were further employed to verify the trigging effect of illumination and ABA on the initiation and progression of leaf senescence in 2014. The full extended flag leaves were carefully detached to conduct an experiment of artificially induced senescence. The flag leaves from two genotypes at that time remained young green, and no visual symptom of leaf senescence had initially emerged for the *psf* mutant. The detached leaves of each genotype were classified into three groups and cut into 3 cm segments to impose different treatments (illumination, darkness and ABA). The detached leaves were smoothed on a wet filter paper and covered with a layer of bright film to keep fresh. For the illumination and darkness groups, The detached leaf segments were floated on 25 mL distilled water in petri dishes, and two artificial growth chamber (Model PRX-450D; Safu, China) were used to layup these petri dishes, with light intensity of 1200 μmol m^-2^ s^-1^ and complete dark condition at 25°C being imposed for illumination and darkness treatments, respectively. For ABA treatment group, the detached leaf segments were floated on solution containing 25mL 250 μM ABA in petri dishes, and then transferred them into the same growth chamber that designed a completely dark condition for the darkness treatment. All detached leaf segments of three treated groups were subsequently sampled at 0 d, 3 d and 6 d after the initial incubation, and the senescence symptoms were taken picture using a digital camera.

To clarify the effect of exogenous ABA concentrations on the transcriptional expressions of various genes involved in D1 protein turnover with respective to leaf senescence, the detached leaf segments of *psf* mutant were further imposed to different ABA concentrations in 2015. The gradient concentration of exogenous ABA was 0 μM, 100 μM, 250 μM, 500 μM and 750 μM, respectively. For each treatment, 25 mL of ABA solution was added in Petri dishes, with 4 dishes for each incubating concentration. Prior to immersion, the leaf segments were placed in distilled water for 2 h to eliminate the wound stress. After 6 d incubation at 25°C in darkness, the leave segments were sampled for the subsequent analysis;

### Determination of net photosynthetic rate (*P*n), chlorophyll content, and chlorophyll fluorescence in rice leaves

Net photosynthetic rate (Pn) were determined by measuring the flag leaf at the time of 09:00–11:00 am, with a LI-6400 portable photosynthesis system (Li-Cor Inc. USA) at constant CO_2_ of 380 μmol in the sample chamber and light intensity of 1200μmol m^-2^s^-1^. The chlorophyll contents (including Chl a, Chl *b* and carotenoid) were assayed as the method described by [36], using a Shimadzu UV-vis 2450/2550 spectrophotometer (Shimadzu, Japan) in three wavelengths (665, 649 and 470 nm). Chlorophyll fluorescence was determined using a portable chlorophyll fluorometer PAM-2000 (WALZ, Germany). The minimal fluorescence intensity (F_o_) in a dark-adapted state was measured in the presence of a background far-red light to favor rapid oxidation of intersystem electron carriers. The maximal fluorescence intensities in the dark-adapted state (F_m_) were determined by 0.8 s saturating pulses (6000 μmol m^-2^s^-1^). The maximal fluorescence level under illumination (F′_m_) was measured by a 0.8 s saturating pulses (6000 μmol m^-2^s^-1^). Variable fluorescence (F_v_) was obtained by subtracting the initial Chl fluorescence (F_o_) from the maximum yield of fluorescence (F_m_). Non-photochemical quenching (NPQ) was calculated from (F_m_/F′_m_) -1. For all these parameters described above, six flag leaves were assayed for each measurement.

### Measurement of ABA concentration, soluble sugar, sucrose, superoxide radical (O_2_^•−^), hydrogen peroxide (H_2_O_2_) and malondialdehyde (MDA) content in rice leaves

ABA in flag leaves was extracted and purified according to the method of [37] with a slight modification. One gram aliquot of leaf sample was homogenized in liquid nitrogen and added to 5ml of frozen extraction buffer (methanol: formic acid: water = 15: 1: 4). After 24 h extraction at -20°C, the extractions were separated by centrifugation (10000 × g, 15min). The supernatant was collected in a 96-well collection plate and the precipitation repeated extracted once again. The two supernatants were merged and collected in 96-well plates.

ABA content was determined by UPLC-ESI-qMS/MS as the method of [37]. After extraction, Oasis HLB96 well collection plate containing the sample was placed in the automatic extraction of solid phase extraction system (SPE215; Gilson, Middleton, WI, USA), the extraction was activated with 1 mol.L^-1^ formic acid before through the column. ABA was washed out by methanol, the eluent was evaporated and further purified. The analysis was carried out on a UPLC-ESI-qMS/MS system. Chromatographic conditions and MS parameters were designed as described by [37]. Triplicate measurements were assayed for each sample.

Soluble sugar and sucrose contents in the leaves were detected as described previously [10,32]. Superoxide radical (O_2_^•−^) generation rate and hydrogen peroxide (H_2_O_2_) content were determined as the method of [31]. Malondialdehyde (MDA) content was assayed according to the method described by [36]. The relative conductivity (RC) was measured as described by [38]. Triplicate measurements were assayed for each sample.

### Extraction of thylakoid membrane protein, SDS-PAGE analysis and Western blotting

The thylakoid membrane proteins were extracted from flag leaf tissues as described by [21] with some modifications. One gram of fresh flag leaves without midrib was well cut into pieces and homogenized with a cooled mortar and pestle in 4 ml of the cool extraction buffer (25 mM Tricine-KOH at pH 7.8, 5 mM EDTA, 5mM MgCl_2_, 0.3 mM sorbitol, 0.1% BSA and 2%(w/v) polycinyl pyrrolidone(PVP)). The homogenate was flltered using 4 layers of cheesecloth to remove large debris, the filtrate was centrifuged at 4,000 × g for 10 min in 4°C. The precipitate was suspended in a hypotonic buffer (25 mM Tricine-KOH at (pH 7.8), 10 mM KOH, 5 mM MgCl_2_, 10% glycerin), and then adjusted the concentration of chlorophyll to 1 mg/ml.

SDS-PAGE analysis for the polypeptides of thylakoid membranes was performed following the method described by [39]. The centrifugal thylakoid membrane of 100 μL was diluted by an equal volume of sample buffer (25 mmol L^−1^ Tris-HCl (pH 6.8), 2% SDS, 5% 2-mercaptoethamol, 10% glycerol, 0.1% bromophenol blue). The mixture was heated at 50°C for 30 min and centrifuged at 10,000 × g for 5 min, and then the supernatant sample of 35 μL was used for SDS-PAGE with 4% stacking gel and 12% separating gel. After finishing electrophoresis, SDS-PAGE gel was transferred to PVDF membranes to detect D1 protein by Western blotting. The procedure of Western blotting was performed according to [40]. The derivatized proteins were sequentially reacted with chicken anti-chloroplast D1 protein (Agresera, AS01-016) and horseradish peroxidase-conjugated rabbit anti-chicken IgG antibodies (Sigma) and visualized by DAB chromogenic kit (Amersham Biosciences).

### RNA isolation, cDNA preparation and Quantitative real-time PCR

The procedures of RNA extraction and cDNA preparation for leaf tissues were performed as described by [35]. Trizol reagent (Invitrogen,Carlsbad, CA, USA) was used for the extraction of total RNA in flag leaves, the ReverTra Ace qPCR RT Kit (TOYOBO, OSAKA, JAPAN) was used for cDNA synthesis as the protocol provided by the manufacturer.

Quantitative real-time PCR were performed by using the SYBR Green Real-time PCR Master Mix reagent Kit (TOYOBO, OSAKA, JAPAN). Reactions were performed on the Bio-Rad CFX96 real-time system (Bio-Rad, USA) following the protocol provided by the manufacturer. The amplification reagents contained 10 μL SYBR, 1.6 μL 10 mM primer pairs, 1 μL cDNA and H_2_O to 20μL. The gene-specific primer pairs used in this study were listed in S1 Table. The *Actin* (X16280) gene was performed as an internal control. The amplification of various genes were normalized by *ACTIN-1* expression and their relative expression levels were determined by the 2^(-ΔΔCT)^ method [41]. The mean values and standard deviation all were measured from triplicate independent biological replicates.

### Statistical analysis

Data analysis was performed using Excel 2003 software. Statistical differences were analyzed by analysis of variance (ANOVA) using the SPSS statistical software package (Chicago, USA). The mean were compared by the least significant difference (LSD) test (*p*<0.05). Standard deviation (SD) was calculated and shown in the figures and tables.

## Results

### Mutant phenotype in the *psf* and its alteration in the senescence-related physiological parameters during leaf senescence

**Fig 1:**
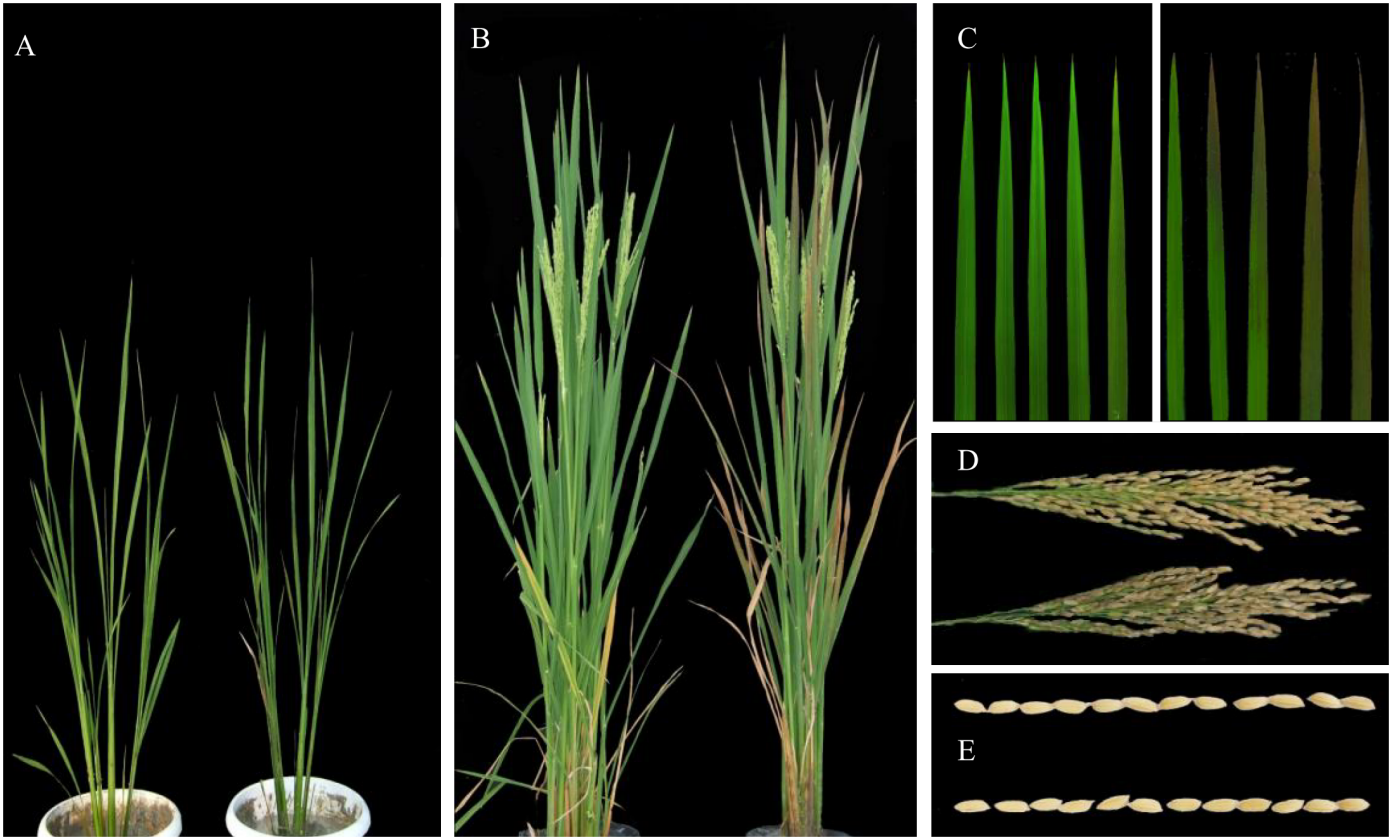
Comparison of plant morphological phenotypes between *psf* mutant and its wild type (Zhehui7954) at different growth stages, and the senescence of flag leaves after anthesis. (A) Tillering stage, left: wild type and right: *psf* mutant. (B) Grain filling stage, left: wild type and right: *psf* mutant (C) The senescent process of flag leaves for *psf* mutant (right) and wild type (left) from anthesis to 28 days after anthesis. (D) The panicle phenotype at mature stage, wild type (above) and *psf* mutant (below). (E) Grain shape, wild type (above) and *psf* mutant (below).

The *psf* mutant exhibited normal growth and development in the seedling stage, no visible differences in plant height, tiller number and leaf appearance (shape and color) were also observed between the *psf* mutant and the wild type at the early and late tilling stages (Fig 1A). Leaf senescence lesions appeared initially on the lower functional leaves of the *psf* mutant after the early booting stage, and the exacerbated lesions subsequently extended to the upper leaves. However, the top-most 1–2 leaves still retained their normal green until the late booting stage (Fig 1B). The flag leaf of *psf* exhibited senescence symptoms post anthesis, and the lesions first appeared on the leaf tip and then spread gradually downward to cover the whole leaf blade and the leaf sheath. Afterward, the flag leaf of *psf* was completely withered approximately at 30 days post anthesis. By contrast, the leaves in the same position of the wild type remained green during the same period (Fig 1B and 1C). Moreover, the *psf* mutant differed evidently from its wild type in seed setting rate within rice panicle (Fig 1D), but was similar to its wild type in grain shape (Fig 1E).

**Fig 2:**
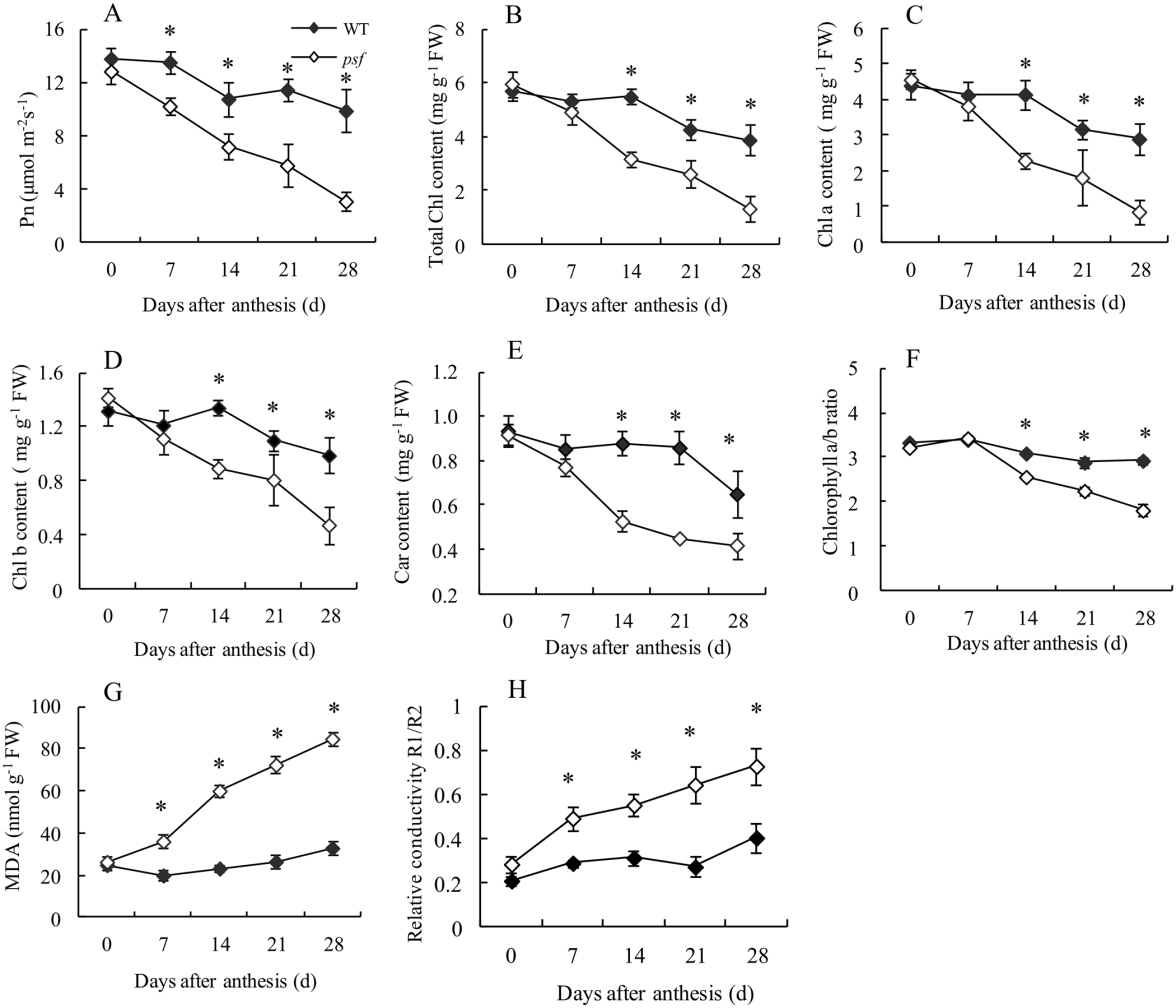
Genotypic differences in the senescent-related physiological parameters and their temporal patterns in the flag leaves of the wild type and the *psf* mutant after anthesis. (A) Photosynthetic rate, (B) The total Chl content, (C) Chl *a* content, (D) Chl *b* content, (E) Carotenoid content, (F) Chlorophyll *a*/*b* rate, (G) MDA, (H) Relative conductivity. Error bars represent standard deviation (*n* = 3). * indicates significant difference (*P* < 0.05) between the wild type and the *psf* mutant.

Photosynthetic rate (Pn), pigment content (Chl and carotenoid), MDA accumulation and relative conductivity (RC) were determined to compare their senescence-associated changes in the flag leaves of the *psf* mutant and wild type after anthesis. As shown in Fig 2, no significant difference in Pn, pigment content, MDA and RC was found between the *psf* mutant and the wild type on the heading day (0 day after anthesis). However, the *psf* mutant showed considerably lower in Pn and photosynthetic pigment (Total Chl, Chl *a*, Chl *b* and Car) content than its wild type from 7 days to 28 days post anthesis (Fig 2A–2E). During this period, Pn, Chl *a* and Car in the *psf* leaves decreased to 30–50% of their maximum levels approximately on the 14^th^ day post anthesis (Fig 2A, 2C and 2E). The Chl *a*/*b* ratio in the *psf* leaf was relatively lower than that in the wild type, with the highest value on the heading day and a marked decline observed for *psf* leaves from 14 days to 28 days post anthesis (Fig 2F). This result implied that Chl *a* degraded more rapidly than Chl *b* during leaf senescence. MDA and RC in *psf* leaves increased rapidly after anthesis, but this phenomenon was not observed in the wild type. The wild type remained a stable level in RC and a slight increase in MDA level throughout the filling stage (Fig 2G and 2H).

**Fig 3:**
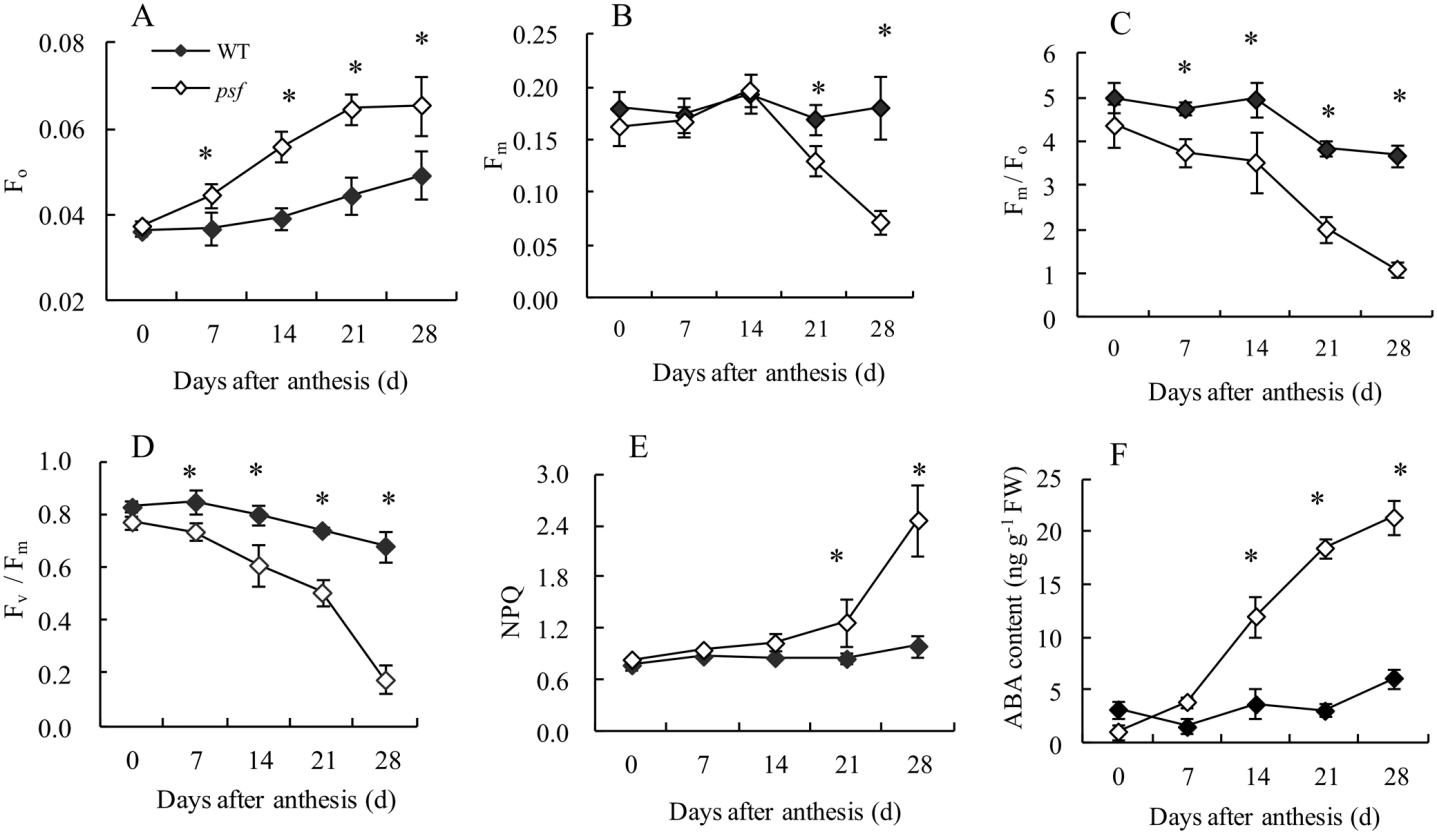
Temporal patterns of chlorophyll fluorescence and ABA content in flag leaves after 0 day to 28 days post anthesis between the wild type and the *psf* mutant. (A) F_0_, (B) F_m_, (C) F_m_/F_0_, (D) F_v_/F_m_, (E) NPQ, (F) ABA content. Vertical bars represent standard deviation (*n* = 3). * indicates significant difference (*P* < 0.05) between the wild type and the *psf* mutant.

Chl fluorescence was further measured to assess the senescence-associated change in the photosynthetic efficiency and electron transfer of PSII reaction center for the two genotypes (Fig 3A–3E). The marked difference in Chl fluorescence parameters (F_o_, F_m_, F_m_/F_o_, F_v_/F_m_ and NPQ) was observed between the *psf* mutant and the wild type, with a substantial increase in F_o_ value for the *psf* leaves after anthesis. The F_m_/F_o_ ratio in the *psf* leaves was significantly lower than that in the wild type from 7 days to 28 days post anthesis. These values were basically similar to the genotype-dependent difference and the temporal pattern in F_v_/F_m_ ratio (Fig 3C and 3D). Furthermore, the senescent leaves of *psf* mutant showed significantly higher NPQ than those of the wild type (Fig 3E). These results indict that the premature leaf senescence of the *psf* mutant may also be characterized by the sharply decreasing electron transfer efficiency in PSII reaction center and the seriously suppressed PSII activity, as reflected by a drastic decrease in F_m_/F_o_ and F_v_/F_m_ for the senescing flag leaves of the *psf* mutant.

A notable difference was found in ABA concentration and temporal patterns between the two genotypes during flag leaf senescence (Fig 3F). The ABA concentrations in the *psf* leaves increased markedly from 7 days to 28 days post anthesis, whereas those in the wild type remained relatively stable or slightly increased over the whole sampling stage. By contrast, the *psf* mutant exhibited a substantially higher ABA concentration in the flag leaves in comparison with the wild type from 7 days to 28 days post anthesis, although the ABA concentration in *psf* leaves was detectable at a low level on the heading day (0 day after anthesis). These results suggested that the genotype-dependent alteration in the timing of senescence initiation and in the subsequent rate of leaf senescence was closely associated with their varying levels in ABA concentrations in rice leaves, because of the marked alteration in their temporal patterns during leaf senescence between the two genotypes. Considering the timing of leaf senescence phenotype and the altering pattern in other senescence-related physiological parameters together, we proposed that the drastic enhancement in endogenous ABA concentration in the *psf* leaf was among the important causative factors for the induction of sunlight-dependent leaf senescence.

### Linking the light-regulated leaf senescence with the varying ABA concentrations

**Fig 4:**
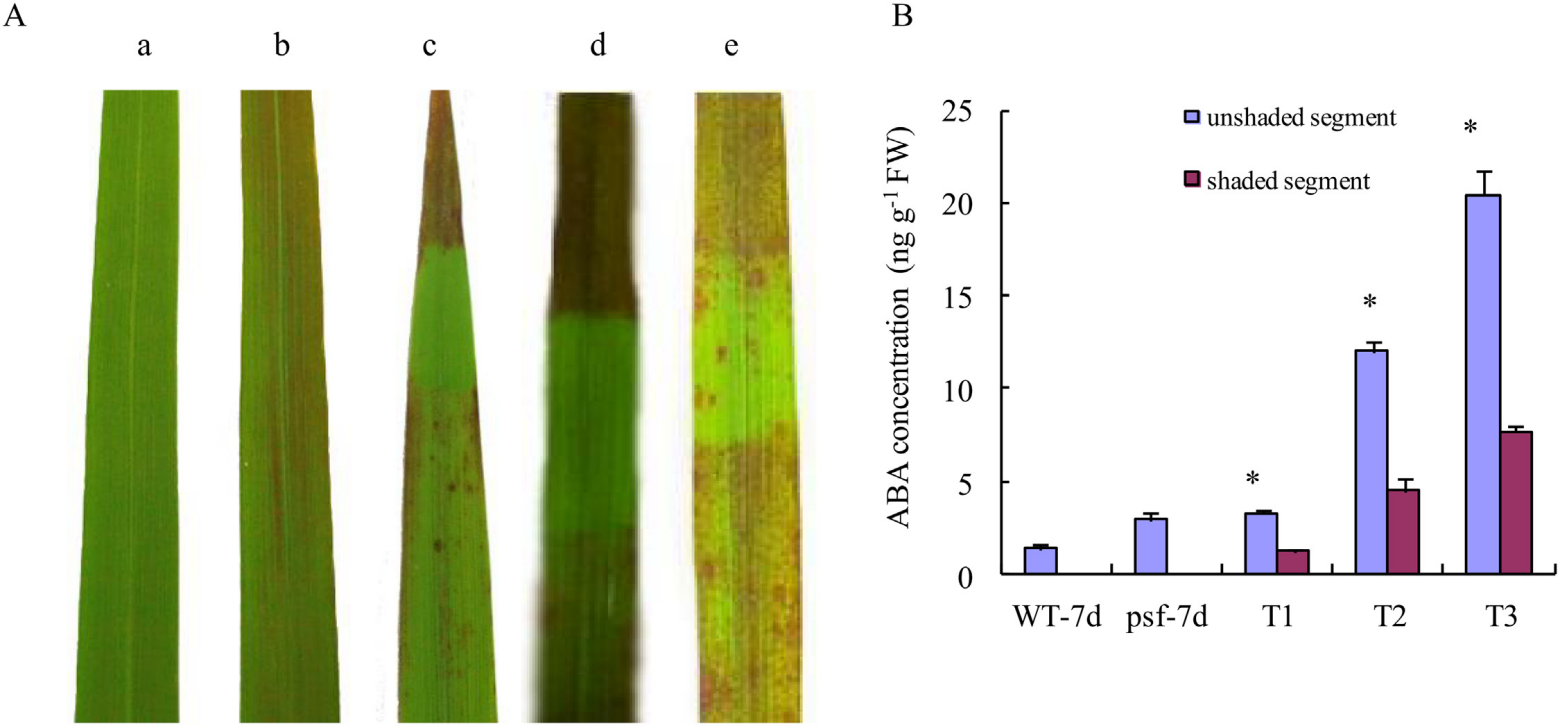
Effects of shaded treatments on the initiation and progression of leaf senescence under natural field condition and its relation to the ABA contents. (A) Visual color and senescent symptom of the flag leaves of the *psf* mutant and it wild type. (A(a)) Flag leaf of wild type at 7 days after anthesis. (A(b)) Flag leaf of *psf* at 7 days after anthesis. (A(c)) Shaded treatment of *psf* flag leaves before the emergence of senescence lesions (Treatment 1). (A(d)) Shaded treatment of *psf* flag leaves after the emergence of senescence lesions (Treatment 2). (A(e)) Re-exposed treatment of the *psf* flag leaves after shaded treatment (Treatment 3). (B) The ABA contents in different flag leaf segments after shaded treatments. WT-7d indicates wild type at 7 days after anthesis. *psf*-7d indicates *psf* at 7 days after anthesis. T1, T2 and T3 represent Treatment 1, Treatment 2 and Treatment 3, respectively. Vertical bars represent standard deviation (*n* = 3). * indicates significant difference (*P* < 0.05).

The symptom and extent of leaf senescence of the *psf* mutant appeared to be sunlight dependent under natural field growth conditions. As shown in Fig 4A, the evidently visible difference in senescent appearance was observed between the shaded area and the unshaded areas within an intact leaf, and the delayed senescence phenotype occurred in the shaded area of the flag leaf. In the shading treatment before the initial emergence of leaf senescence symptoms (Treatment 1), the shaded area remained almost normal green, and the rust-like spots appeared only in the unshaded area of the flag leaf (Fig 4A(c)). Interestingly, the occurrence of rust-like spots, which reflected the premature senescence phenotype, could be alleviated by the shading treatment after the early emergence of leaf senescence symptoms (Treatment 2), and the visually reduced density of rust-like spots and even the disappearance of rust-like spots were observed in the shaded area (Fig 4A(d)). Comparatively, when the shaded area was re-exposed to sunlight for seven days of solar irradiation, premature rust-like spots appeared again (Fig 4A(e)). These results illustrated that the appearance of premature rust-like spots in the *psf* mutant leaf was sunlight-dependent under field growth condition.

To investigate whether or not the alteration in the sunlight-dependent leaf senescence phenotype was regulated by its possibly varying ABA concentrations among different leaf areas, we verified the endogenous ABA concentration in the treated flag leaves, with the different areas being discriminated for an intact leaf (Fig 4B). A strikingly significant difference in ABA concentration was observed between the shaded and the unshaded area within a flag leaf. In Treatment 1, the ABA concentration of the unshaded areas was 2.7 fold higher than that of the shaded area. Moreover, the re-exposure of the shaded area to sunlight caused a drastic increase in ABA concentration in this area, but the ABA concentration in the re-exposed area remained considerably lower than that in the unshaded area (Treatment 3). Considering the genotype-dependent alteration in endogenous ABA concentration and their temporal pattern during leaf senescence (Fig 3F) together, we deduced that the effect of sunlight on the leaf senescence appearance was closely related to the endogenous ABA concentration in the *psf* leaves, with the accelerated senescence symptom for higher ABA level in the flag leaves induced by sunlight.

**Fig 5:**
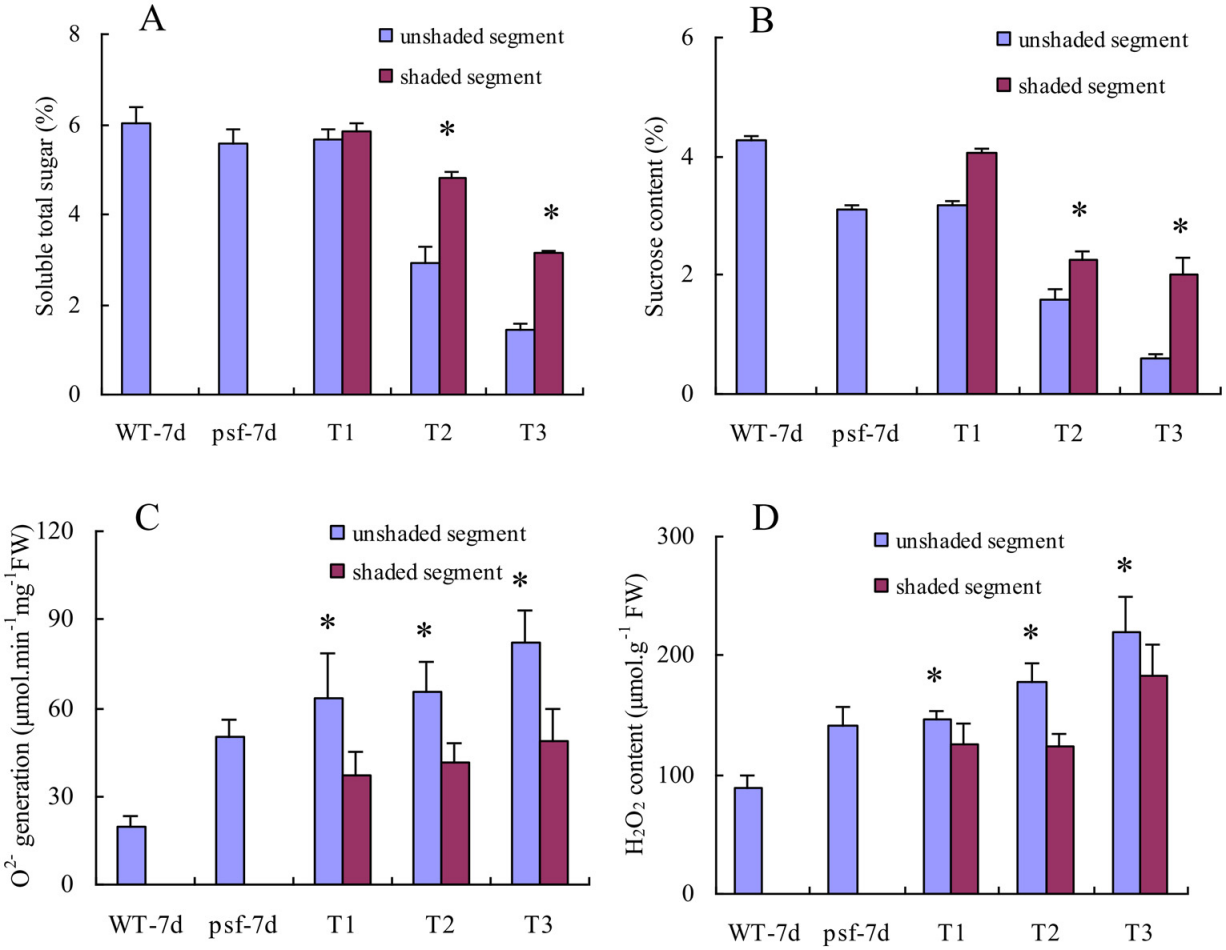
Effect of shaded treatments on soluble total sugar content (A), sucrose content (B), O2•− generation rate (C) and H_2_O_2_ content (D) in flag leaves under natural field condition. WT-7d indicates wild type at 7 days after anthesis. *psf*-7d indicates *psf* at 7 days after anthesis. T1, T2 and T3 represent Treatment 1, Treatment 2 and Treatment 3, respectively. Vertical bars represent standard deviation (*n* = 3). * indicates significant difference (*P* < 0.05).

We compared the difference in soluble total sugar, sucrose content, O_2_^•-^ generation rate and H_2_O_2_ content between the shaded and the un-shaded areas for the different treatments (Fig 5). In Treatment 1, the shaded area didn’t evidently differ from the un-shaded area in soluble total sugar and sucrose content (Fig 5A and 5B), but the former showed significantly lower levels of O_2_^•-^ generation rate and H_2_O_2_ accumulation than the latter (Fig 5C and 5D). In Treatment 2 and Treatment 3, the contents of soluble total sugar and sucrose in the shaded area were significantly higher than those in the un-shaded areas (Fig 5A and 5B), whereas it was opposite for O_2_^•-^ generation rate and H_2_O_2_ content, the un-shaded area had the significantly higher O_2_^•-^ generation rate and H_2_O_2_ content than the shaded area (Fig 5C and 5D).

**Fig 6:**
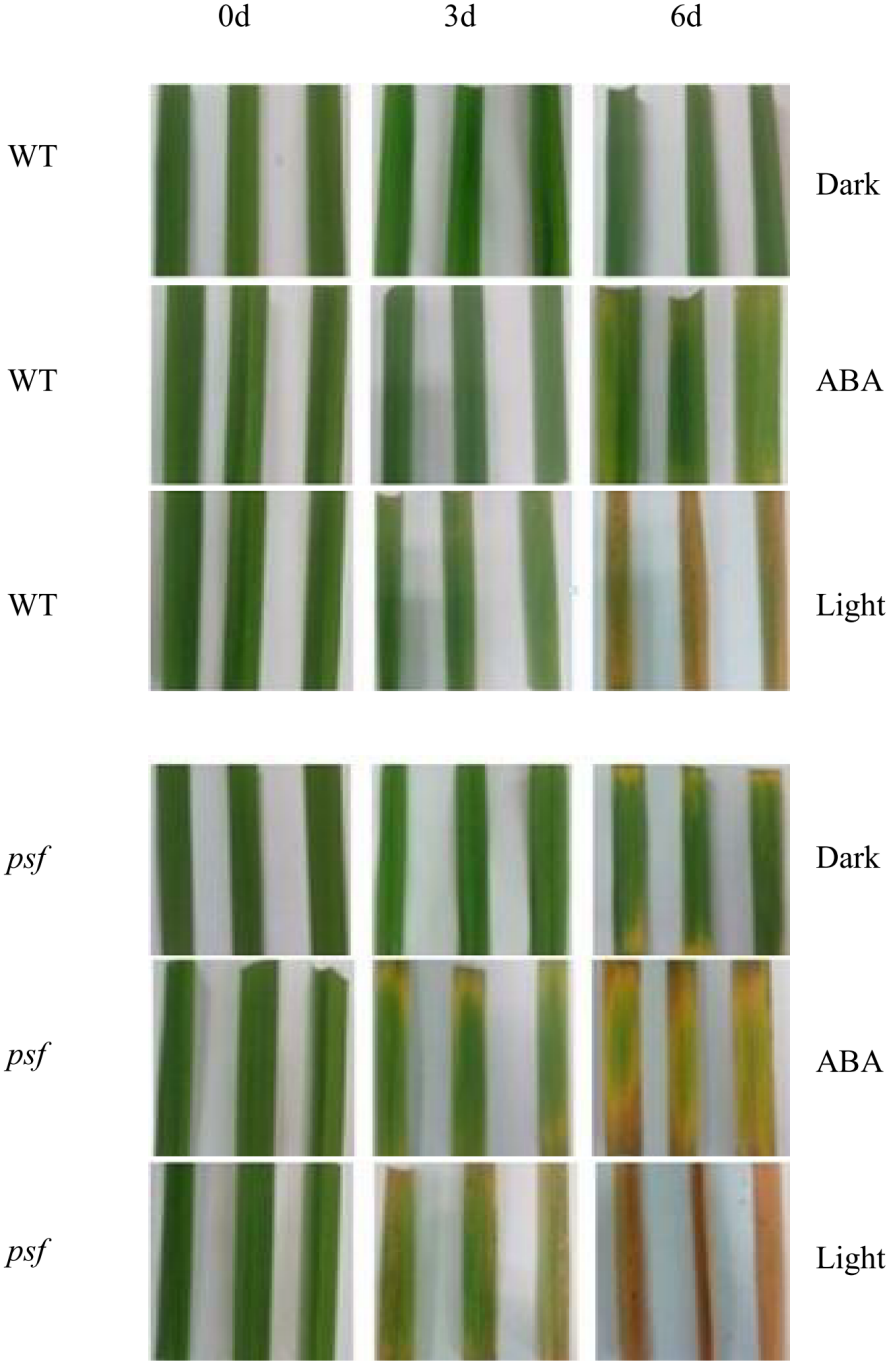
Visual color and senescent symptom of detached leaf segments induced by exogenous ABA incubation (250 μM concentration) at 0d, 3d and 6d, respectively. Similar immersions with the distilled water under the light intensity of 1200 μmol m^-2^ s^-1^ and completely dark conditions were implemented as controls. The upper is wild type (WT), the downer is the *psf* mutant (*psf*).

**Fig 7:**
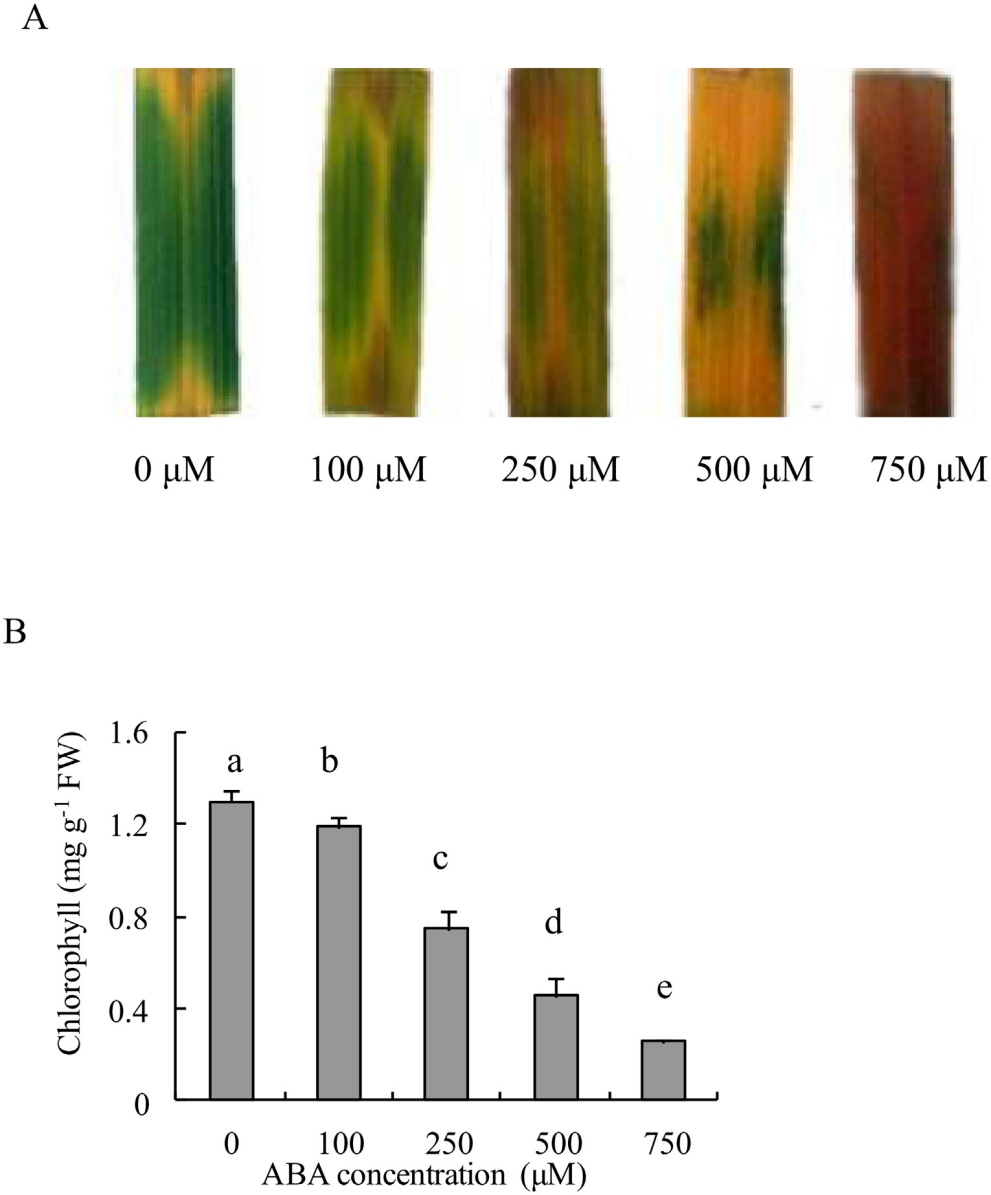
Senescent symptoms and total chlorophyll content changes of detached leaf segments induced by a gradient concentrations of exogenous ABA (0 μM, 100 μM, 250 μM, 500 μM and 750 μM) after 6 days incubation, respectively. (A) Visual color change of detached leaf segments. (B) Total chlorophyll contents in detached leaf segments after 6 days incubation. Vertical bars represent standard deviation (*n* = 3). Different letters indicate significant differences (*P* < 0.05).

To further confirm the motivating effect of ABA on light-dependent leaf senescence, the detached leaf segments were employed to impose exogenous ABA treatments, and distilled water immersion under illumination and that in darkness conditions were used as two controls (Fig 6). Similar to illumination in the incubated chamber, exogenous ABA incubation resulted in fading leaf green, chlorosis appearance, and decreasing Chl content after three to six days of treatment, and the extent of decreasing Chl content was dependent on different incubating durations and rice genotypes. Detached leaf segments of the *psf* mutant appeared to be more sensitive to exogenous ABA incubation than those of the wild type (Fig 6 and S2 Table). Under our experimental conditions, the illumination appeared to exert a more profound impact on senescing appearance and Chl content than the ABA incubation of 250 μM concentration. Accordingly, the detached leaf segments of the *psf* mutant were further imposed to a series of gradient ABA concentrations (0,100, 250, 500, and 750 μM). The result indicted that leaf senescence was accelerated by increasing ABA concentration. Severely exacerbated chlorosis appearance and apparently decreased Chl content were observed for flag leaf segments treated by higher ABA concentrations (500 and 750 μM) (Fig 7).

### Relationship of ABA-induced premature leaf senescence with D1 protein damage of PSII reaction center in chloroplasts

**Fig 8:**
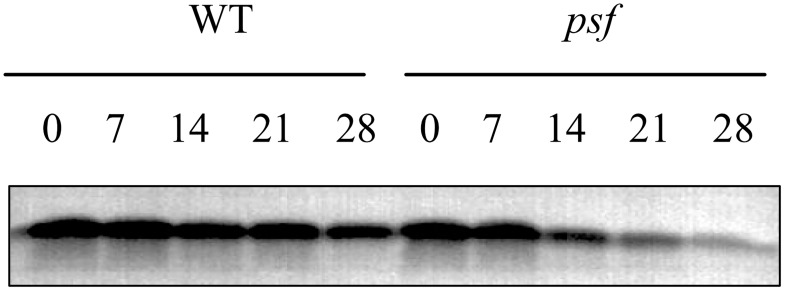
Genotypic differences in D1 protein abundance detected by Western blotting and their temporal pattern in the flag leaves of wild type and the *psf* mutant after anthesis. 0, 7, 14, 21 and 28 indicate days after anthesis.

The D1 protein in the PSII reaction center is the major target of photodamage among all the thylakoid proteins for the plants exposed to stressful environments, and the abnormal degradation of D1 protein results in the loss of PSII activity and Chl content in plant leaves, along with decreasing photosynthetic rate [16,18]. To examine the association of ABA accumulation in light-dependent premature leaf senescence with the degradation and photodamage of D1 protein in the PSII reaction center, we detected the genotype–dependent alteration in D1 protein abundance in the senescing leaves for the *psf* mutant and its wild type by Western blotting. The result showed that the D1 protein levels in the *psf* flag leaf declined rapidly from 7 days to 28 days post anthesis; the lowest D1 protein amount was detectable on the 28^th^ day post anthesis, whereas that in the wild type declined gradually until rice harvest (Fig 8). Interestingly, the decline in D1 protein levels and their temporal pattern during leaf senescence were concomitant with the increase in ABA concentration and decrease in PSII activity in *psf* leaves, as reflected by the Chl fluorescence parameters (F_o_, F_m_, F_m_/F_o_, and F_v_/F_m_) and endogenous ABA determination (Fig 3). Thus, we inferred that the rapid damage/abnormal degradation of D1 protein in the PSII reaction center could be one of the most crucial steps for the linkage between light-dependent leaf senescence and the varying ABA concentrations in *psf* mutant leaves.

**Fig 9:**
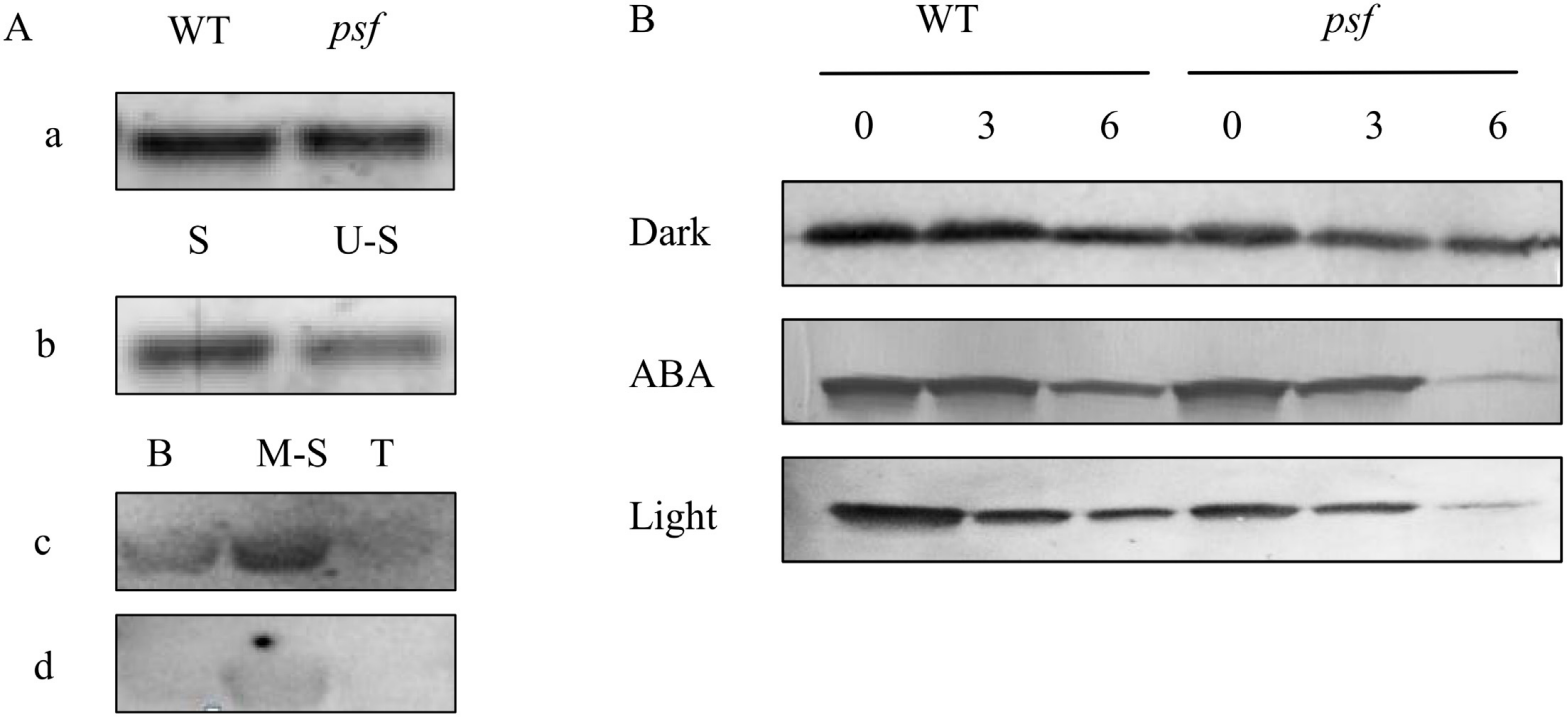
Changes in D1 protein abundance detected by Western blotting for the un-detached and detached leaves of different treatments. (A) Effects of shaded treatments on the D1 protein abundance detected by Western blotting. (A(a)) D1 protein amount in flag leaves of Fig 4A(a) and 4A(b). (A(b)) D1 protein amount in different leaf segments of Fig 4A(c), S: indicates shaded area, U-S: indicates un-shaded area. (A(c)) D1 protein amount in different leaf segments of Fig 4A(d), B: indicates base un-shaded area, M-S: indicates middle shaded area, T: indicates tips un-shaded area. (A(d)) D1 protein amount in different leaf segments of Fig 4A(e). (B) Effects of ABA, dark and light treatments on the D1 protein abundance detected by Western blotting in detached leaf segments of wild type and *psf* mutant. 0, 3 and 6 indicate days after incubation.

The difference in D1 protein level between shaded and unshaded areas under the shaded field treatments was further determined to investigate the effect of sunlight on D1 protein level with respect to ABA-induced leaf senescence. As shown in Fig 9, the D1 protein amount in the shaded area was markedly higher than that in the unshaded area after seven days of solar irradiation (Fig 9A(b) and 9A(c)). Furthermore, the re-exposure of the shaded area to sunlight appeared to decrease in D1 protein abundance in the area, although the D1 protein level in the re-exposure area was still higher than that in the unshaded area (Fig 9A(d)). These results implied that the delayed leaf senescence in the shaded areas could be attributable to the inhibited D1 protein degradation accompanied by decreasing ABA concentration and delayed leaf appearance in the shaded areas. In addition, when the detached flag leaf segments were subjected to exogenous ABA incubation or imposed to the illumination in light chamber, the D1 protein levels in the detached leaf segments evidently decreased, with an extremely low level of D1 protein for a six-day incubation duration (Fig 9B). Comparatively, only a slight decline in D1 protein amount was observed for the detached segments treated by the distilled water in darkness after a six-day incubation (Fig 9B). These phenomena demonstrated that exogenous ABA incubation could impair D1 protein biosynthesis, or exert a motivating effect on the damaged process of D1 protein.

### Transcriptional expression of key genes involved in D1 protein turnover and PSII repair cycle in relation to ABA-induced leaf senescence

D1 protein has the highest turnover rate among the thylakoid proteins [42], and this turnover plays an indispensible role in the repair cycle of PSII reaction center and the maintenance of Chl-protein complexes located in the thylakoid membrane [43,44]. To explore the interplay between the possibly altered D1 protein turnover and ABA-induced leaf senescence for the *psf* mutant, we comprehensively investigated the transcriptional expression of several key genes that participate in D1 protein turnover and PSII repair cycle by using quantitative real-time reverse transcription PCR.

**Fig 10:**
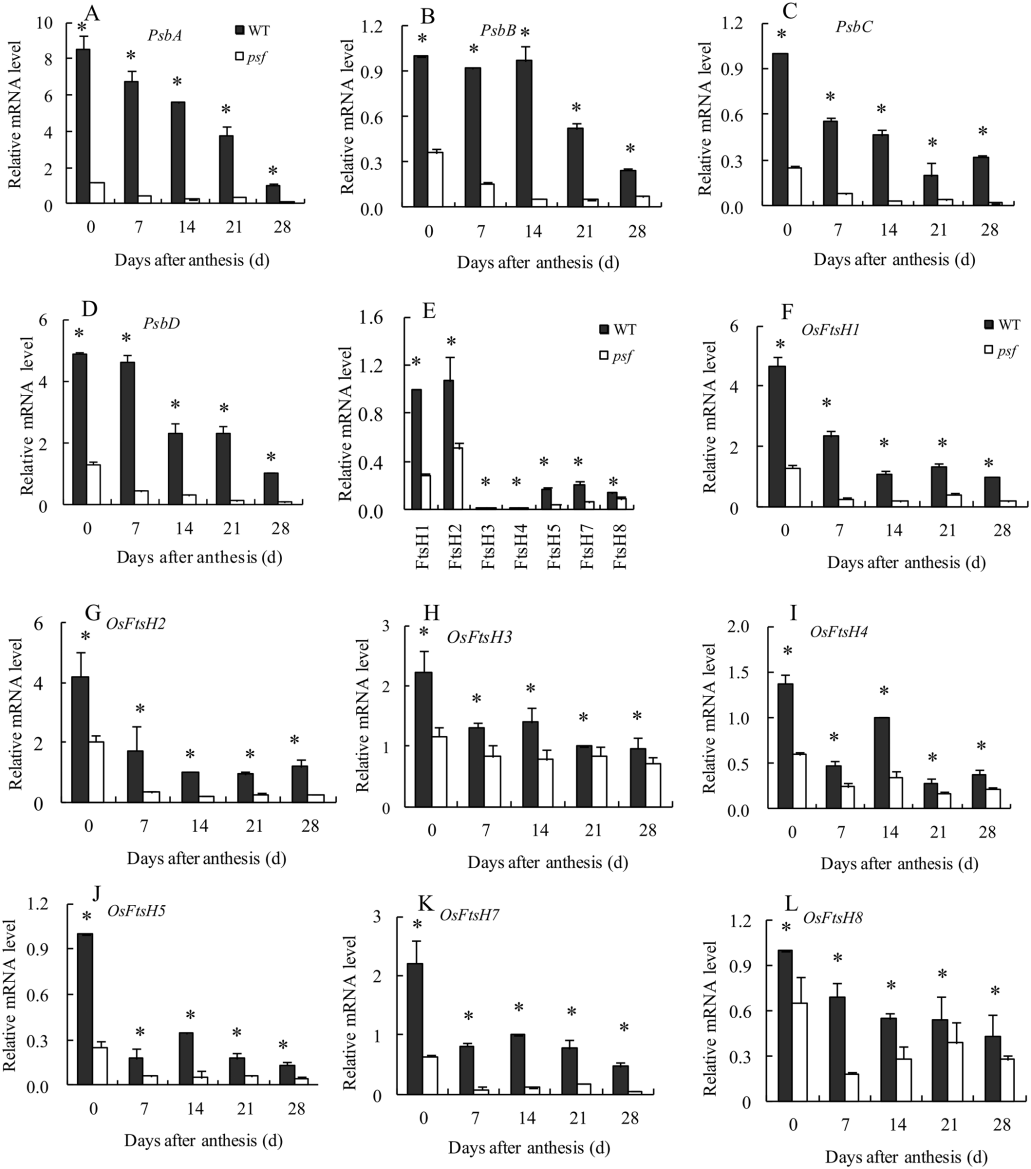
Genotypic difference in the mRNA transcript levels of several key genes involved in D1 protein turnover and their temporal patterns in the flag leaves of wild type and *psf* mutant after anthesis. (A) *PsbA*, (B) *PsbB*, (C) *PsbC*, (D) *PsbD*, (E) Comparison of seven FtsH isoform genes expressions at 0 day after anthesis. (F) *OsFtsH1*, (G) *OsFtsH2*, (H) *OsFtsH3*, (I) *OsFtsH4*, (J) *OsFtsH5*, (K) *OsFtsH7*, (L) *OsFtsH8*. Vertical bars represent standard deviation (*n* = 3). * indicates significant difference (*P* < 0.05).

As shown in Fig 10, a significant difference in the transcript level of *PsbA*, which modulates the de novo synthesis of D1 protein in thylakoid membrane, was detected between the two genotypes. The flag leaves of the *psf* mutant exhibited a considerably lower level of *PsbA* transcript than those of the wild type during the entire sampling period (Fig 10A). The maximum level of the *PsbA* transcript in flag leaf occurred at anthesis, with a 7.2 fold higher transcript in the wild type than in the *psf* mutant. Subsequently, the transcript of *PsbA* decreased continuously until grain maturity, with virtually no detectable levels observed for the *psf* mutant at 28 days post anthesis (Fig 10A). Similar to *PsbA*, the *psf* mutant differed evidently in the transcripts of *PsbB*, *PsbC* and *PsbD* from the wild type, with significantly lower transcripts for the *psf* mutant than for the wild type (Fig 10B–10D). Among the seven *FtsH* genes that regulate D1 protein degradation, *FtsH1* and *FtsH2* were highly expressed in rice leaves; the transcripts of *FtsH5*, *FtsH7* and *FtsH8* were moderately abundant in the two genotypes; and the transcripts of *FtsH3* and *FtsH4* were detectable at an extremely low level (Fig 10E). Furthermore, the transcript levels of all seven *FtsH* genes in the *psf* leaf were significantly lower than those in the wild type, with a decreased change in temporal pattern during leaf senescence, despite the isoform-specific variation in the extent of their decreasing transcripts for the *psf* mutant (Fig 10). These data suggest that the transcriptional expression of the *FtsH* family genes in rice leaves is important for the implications of D1 protein turnover and the maintenance of PSII activity during leaf senescence. The inhibitory expression of *FtsH* family genes in the *psf* leaves, occurring concomitantly with the significant decrease in *Psb* transcripts, may lead to an impairment of D1 protein turnover during leaf senescence. This result implies that both the inhibition of de novo synthesized D1 protein and the slow rate of proteolytic removal were responsible for the damaged D1 protein, thereby an evident decrease in the D1 protein amount for the *psf* leaves.

**Fig 11:**
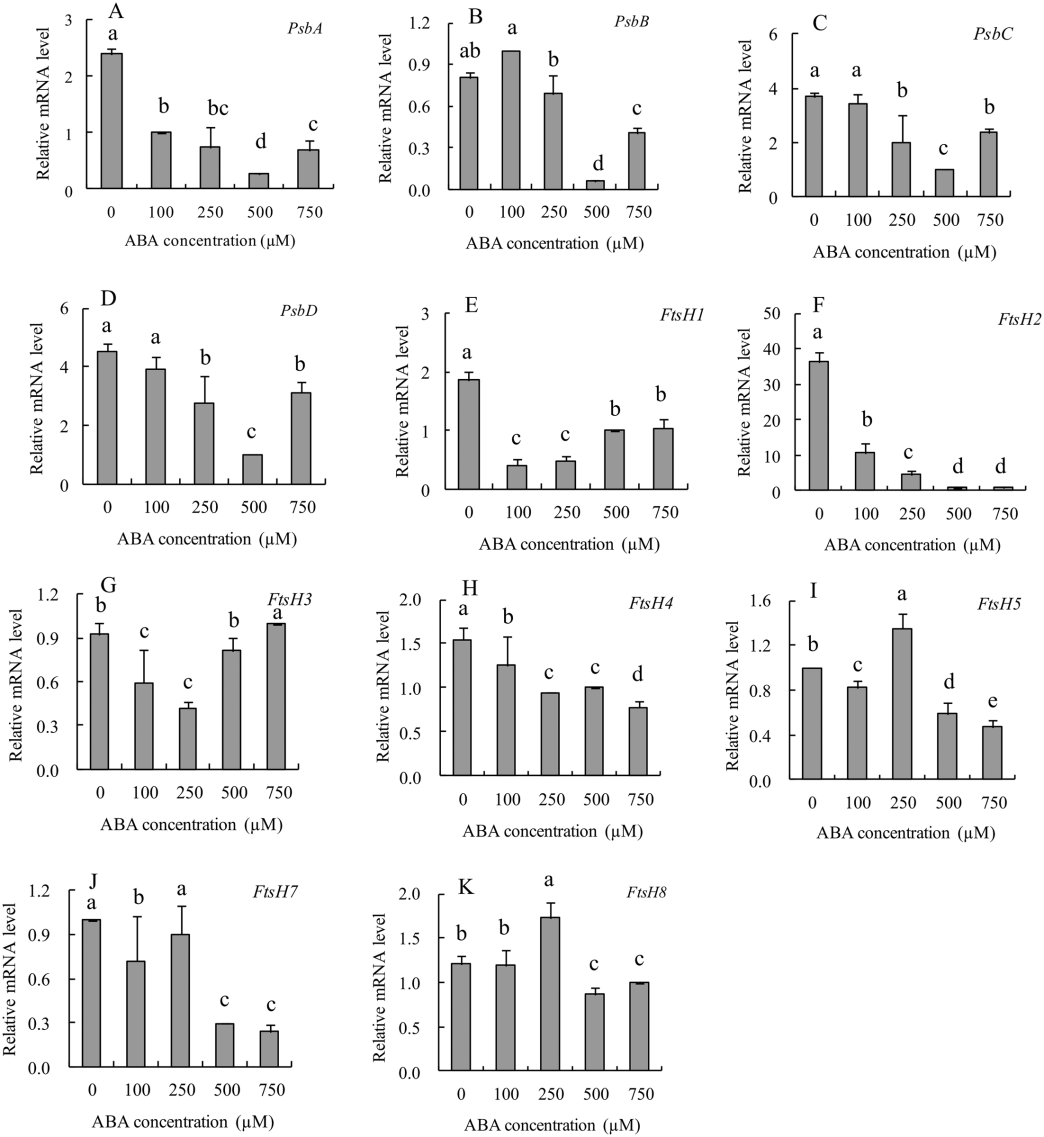
Effects of ABA concentration on the transcript levels of key genes involved in D1 protein turnover in *psf* leaf segments. (A) *PsbA*, (B) *PsbB*, (C) *PsbC*, (D) *PsbD*, (E) *FtsH1*, (F) *FtsH2*, (G) *FtsH3*, (H) *FtsH4*, (I) *FtsH5*, (G) *FtsH7*, (K) *FtsH8*. Vertical bars represent standard deviation (*n* = 3). Different letters indicate significant differences (*P* < 0.05).

The effect of ABA on the transcript levels of *Psb* and *FtsH* genes was examined using the detached leaf segments of the *psf* mutant, with a series of gradient ABA concentration being imposed (Fig 11). The result showed that the transcripts of *PsbA* were significantly downregulated by exogenous ABA, and the *PsbA* level in the detached leaves decreased 2-fold in 100 μM ABA concentration and 10-fold in 500 μM ABA incubation, respectively (Fig 11A). By contrast, no significant difference was detected between 100 μM ABA treatment and their CKs (distilled water incubation) for the transcripts of *PsbB*, *PsbC* and *PsbD*, although the expression of *PsbB*, *PsbC* and *PsbD* also tended to be suppressed by 250 μM and 500 μM exogenous ABA incubations (Fig 11B–11D). These data implied that the suppression of *Psb*A transcript was easily triggered by increasing ABA level in the senescent rice leaves, thus consequently leads to a sharply decreasing de novo synthesis of D1 protein in *psf* flag leaves. By contrast, the function of other genes (*PsbB*, *PsbC* and *PsbD*) was evidently impaired only with the accumulation of a certain high level of ABA concentration in rice leaves. Furthermore, the effect of exogenous ABA on *FtsHs* transcripts was considerably variable, depending on the *FtsHs* isoforms and ABA concentration (Fig 11E–11K). For instance, the *FtsH2* transcript was markedly repressed by exogenous ABA (Fig 11F), and a significant decrease in the transcripts of *FtsH1* and *FtsH3* was observed only in low ABA concentrations (100 and 250 μM), with elevated transcripts of *FtsH1* and *FtsH3* in a high ABA concentration (>500 μM) relative to their corresponding CKs (Fig 11E and 11G). In addition, the transcript amounts of *FtsH1* and *FtsH2* were downregulated by 250 μM ABA concentration (Fig 11E and 11F), and similar changes were observed in a genotype-dependent temporal pattern during leaf senescence (Fig 10F and 10G). These results imply that *FtsH2* is one of the most important genes for the regulation of D1 protein turnover and PSII repair cycle in relation to ABA induced leaf senescence, accompanied by the altered *PsbA* transcript that controls the de novo synthesis of D1 protein in the PSII reaction center.

## Discussion

Previous studies demonstrated that light intensity play a significant role in trigging or delaying leaf senescence [18,19,25,45]. Studies on *Arabidopsis thaliana* and other plant species indicated that leaf senescence accelerated significantly under increased light intensity [46], because excessive light energy resulted in the occurrence of photo-inhibition and oxidative damage [19]. On the other hand, the effect of light deprivation and low solar illumination on leaf senescence was investigated by many previous reports [5,18], which concluded that exposure to the shade/darkness often accelerates leaf senescence in many plant species [18,47]. In a densely growing crop population, the shade-induced senescence occurred at increased plant densities for leaves below a canopy [48]. According to [12], the decrease in blue and/or near-ultraviolet region of the light spectra is a key signal for the induction of senescence in shaded leaves under natural conditions. However, recent studies revealed that the induction of leaf senescence depends more on light intensity than on light quality, which is expressed as the red/far-red (R/FR) ratio [18,49]. Under low light intensities, the R/FR ratio enhanced photosynthetic acclimation, but not Chl degradation [18]. In this paper, the premature senescent appearance of *psf* leaves was found to be light-dependent under field growth conditions, and the induction of solar illumination on leaf senescence was closely associated with increasing ABA concentration in *psf* mutant leaves (Fig 4). However, our present study revealed that the deprivation of sunlight for the part areas of individual leaves in growing rice plants exerted an inhibitory effect on the occurrence of leaf senescence under natural field conditions, and that the shaded area differed evidently from the unshaded area in visibly senescing lesions and phenotypes (Fig 4A). This result appears to disagree with several lines of previous studies, which concluded that the darkening/shaded treatment for individual leaves (either in growing plants or in detached segments) induced leaf senescence [5,48,50]. These conflicting results could be explained by the occurrence of “sugar starvation” in shaded leaves, as sugar starvation is widely considered to trigger plant leaf senescence in many cases [51]. Sugar starvation is easily caused by the darkening treatment for a whole plant or an individual full leaf because of the complete loss of leaf photosynthesis, which consequently induces leaf senescence [4,50]. Different from the conventional shading or darkening treatments, our experimental set-up was conducted using an approach in which only a portion of a full leaf was shaded, while the remaining leaf portions were exposed to sunlight. Thus, sugar starvation was effectively alleviated for the shaded areas of a full leaves in growing rice plants, because of the relative sufficient sugar supply along the phloem transport within the same leaf. Indeed, no significant difference in soluble total sugar and sucrose content was detected between the shaded areas and unshaded ones after seven days of shaded treatment (T1), and the re-exposed areas even exhibited significantly lower levels of soluble total sugar and sucrose content than the shaded areas after the emergence of leaf senescence lesion (Fig 5A and 5B). On the other hand, the shaded areas of a full leaves in growing rice plants were protected by the darkness treatment to prevent the occurrence of photoinhibition and oxidative damage, as reflected by the significantly lower levels of O_2_^•-^ generation rate and H_2_O_2_ accumulation in comparison with the unshaded area within the same leaf (Fig 5C and 5D). Consequently, the senescent symptom of rust-like spots appeared only in the unshaded area of rice leaves for the *psf* mutant. Interestingly, the occurrence of rust-like spots could be alleviated and even repaired by a subsequent shading-treatment after the early emergence of the leaf senescence symptom (Fig 4A(d)). This result implies that the participation of shading treatment in regulating leaf senescence for the growing rice plants is closely associated with the recovery process of injury symptom through the repair of the damaged leaf segments, in addition to the inhibition of senescence-associated oxidative damage and protein degradation. Furthermore, previous studies had well demonstrated that the levels of reactive oxygen species (ROS) in plant leaves was strongly responsible for leaf senescence symptom [6,25] and ABA resulted in the increased generation of ROS in plant leaves [34]. Thus, it was not surprisingly found that the flag leaves of the *psf* mutant had significantly higher level of O_2_^•-^ generation and H_2_O_2_ content than those of its wild type under natural field growth conditions (S1 Fig), thereby the significantly enhanced MDA accumulation and RC value being observed for the *psf* mutant leaves (Fig 2G and 2H). The light inducible increase in ABA concentration (Fig 4B) appeared to be closely associated with the significant enhancement in ROS generation in the sunlight-exposed areas (Fig 5C and 5D). However, the oxidative stress and ROS generation in plant organs could be also activated by ABA-independent pathway [6,22]. Thus, further study is required to understand the interaction between ROS and ABA metabolism in respective to sunlight-dependent leaf senescence.

In higher plants, solar energy conversion relies on chloroplasts. Chloroplasts are the most vulnerable organelles to photo-oxidative damage [7]. In chloroplasts, the PSII reaction center D1 protein in the thylakoid membranes of chloroplasts is the originating site of photoinhibition and photodamage [17,52]. Several lines of study revealed that the accumulation of ABA in plant leaves seriously affects the rate of photosynthetic electron transport system and induces the degradation of D1 protein in the thylakoid membrane stacking area. The data presented here indicated that the abnormal accumulation of ABA concentration in the *psf* leaves was strongly responsible for the initiation and progression of leaf senescence, which was sunlight dependent under field growth conditions (Fig 3). The involvement of ABA in light inducible senescence for the *psf* mutant leaves was also illustrated by the considerably lower ABA concentration in the shaded region, where leaf senescence symptom was evidently retarded by darkness in comparison with the unshaded region in intact leaves (Fig 4). Exogenous ABA incubation resulted in the sharply decreasing amount of D1 protein in the detached leaf segments (Fig 9B). According to [10,32], the photosynthesis rate, F_m_/F_o_ value and Chl content in rice leaves were significantly decreased by the exposure of rice plants to severe water deficiency, but water stress increased the ABA levels in rice leaves and induced the occurrence of leaf senescence [10,32]. In our current results, the *psf* mutant evidently differed from the wild type in the initiation and progress of leaf senescence (Figs 1 and 2). Correspondingly, the genotype-dependent alteration in the timing of senescence initiation and in the subsequent rate of leaf senescence was closely associated with the varying ABA concentration levels in rice leaves (Fig 3). The Western blotting result of D1 protein illustrated that the decreased level of D1 protein and its temporal pattern during leaf senescence agreed with the tendency in increasing ABA and lowering the PSII activity in the *psf* mutant leaves (Figs 3 and 8). The D1 protein amount in the shaded area was substantially higher than that in the unshaded area after seven days of solar irradiation (Fig 9A(b) and 9A(c)), and the re-exposure of the shaded area to sunlight also resulted in a rapid decrease in D1 protein content (Fig 9A(d)). In addition, D1 degradation and Chl loss in the detached leaf segments were impelled by exogenous ABA incubation and light (Fig 9B and S2 Table). These results suggested that the delayed leaf senescence in the shaded areas could be attributable to the inhibited D1 protein degradation and the accelerated D1 protein synthesis, accompanied by lowering the ABA concentration in the shaded areas. At the transcriptional level, the suppressed expression of the *Psb*A gene encoded D1 protein was easily triggered by the increasing ABA level in the senescing rice leaf, which in turn resulted in the sharply decrease in de novo synthesis of D1 protein in the *psf* flag leaves. By contrast, the expression of other *Psb* genes (*PsbB*, *PsbC*,and *PsbD*) was also impaired evidently at a certain high level of ABA concentration accumulated in rice leaves (Fig 11). Hence, the acceleration of leaf senescence for *psf* mutant leaves exposed to sunlight was caused not only by the relatively high rate of D1 protein degradation in PSII reaction center, but also by the low rate of D1 protein synthesis in the presence of ABA. Previous studies revealed that Chl degradation and D1 oxidative damage are early senescence signals [15,43], and that the translation of *psbA* mRNA was markedly suppressed by exogenous ABA application [1,30]. Our present result provided the evidence that the PSII reaction center D1 protein suffered photodamage in the presence of increasing ABA levels in the *psf* leaves under solar exposure. Furthermore, the turnover (damage and repair) of D1 protein is closely related to the inactivation and the recovery of PSII activity, as induced by photoinhibition [16,17]. In our present study, the importance of repairing PSII activity through re-synthesis of the D1 protein during light exposure was demonstrated by the recovery increase in D1 protein amount in darkness, accompanied by the decrease in ABA concentration in rice leaves. In previous studies, it had well been documented that the loss of PSII activity for the plant leaves exposed to unfavorable or stressful environmental conditions was closely associated with the slow rate of D1 protein turnover, the inhibition of the repair of photodamaged D1 protein, and degradation of the damaged D1 protein in PSII complex [9]. The repair of PSII is much more sensitive to environmental stress than the process of photodamage to PSII [23]. Exposure for moderate stresses, the rate of D1 protein turnover and PSII repair cycle in plant leaves were evidently inhibited, but the extent of photodamaged D1 protein in PSII didn’t enhance significantly, thus resulting in the reversible photo-oxidative damage to PSII activity [23]. According to [24], moderate salt stress inhibited the repair of photodamaged D1 protein in PSII, but it did not directly accelerate the photo-oxidative damage of PSII function in tobacco leaves. However, our present result suggested that the loss of PSII function during leaf senescence was, at least partly, attributable to the accelerating degradation of photo-damaged D1 protein, although the light inducible ABA enhancement also resulted in the slow rate of D1 protein turnover and the inhibited repair of photodamaged D1 protein in the *psf* leaves (Figs 8 and 10).

The degradation of chloroplast proteins can be implemented by chloroplast proteases within the plastid itself [53], and FtsH proteases may function crucially in the proteolysis of the photodamaged D1 protein and other chloroplast proteins in the PSII reaction center [54,55]. In rice, seven members of a distinct *FtsH* form have been recently identified in this gene family, and the members can be classified into three classes based on sequence alignments and cellular location [22,56]. The protease encoded by FtsH2 and FtsH5 act as a heteromeric complex in the thylakoid membrane, with the ATP-binding domain and catalytic zinc-binding site facing the stroma [53]. Proteases encoded by *FtsH1*, *FtsH6*, and *FtsH7* are also targeted to chloroplasts, whereas those encoded by *FtsH3* and *FtsH 4* are mitochondrial [44,56]. Homology-based analysis results indicate that the FtsH family proteases are highly conservative among different plant species [56]. Studies on the *Arabidopsis* mutant devoid of the *AtFtsH2* gene showed that the loss of *FtsH2* expression resulted in the most severe variegation and sensitivity to photoinhibition under various stress conditions [56]. In this paper, *FtsH2* was highly expressed in rice leaves, and the transcriptional levels of *FtsH1*, *FtsH5*, *FtsH7*, and *FtsH8* were moderately abundant for the two rice genotypes, whereas the transcripts of *FtsH3* and *FtsH4* were detectable at an extremely low level (Fig 10). These results corroborated several previous findings, in which the transcript amounts of genes encoding FtsH proteases that target chloroplasts were more abundant than those targeting mitochondria in *Arabidopsis* [54,55,56]. Furthermore, our experimental data clearly indicted that the transcript levels of all *OsFtsH* genes in the *psf* leaf were significantly lower than those in the wild type, with reduced change in temporal pattern during leaf senescence (Fig 10). These results implied that the suppressed transcripts of *OsFtsH* family genes in the *psf* leaf were also responsible for the rapid decrease in D1 protein amount and PSII activity during leaf senescence. According to [22,55,56], FtsH proteases are involved in PSII repair by the proteolytic removal of photo-damaged D1 protein, followed by the coordinated insertion of the newly synthesized D1 protein into the thylakoid membrane [22,55,56]. Thus, the turnover of D1 protein in the *psf* leaves could be impaired by the rate of slow proteolytic removal for the damaged D1 protein during leaf senescence aside from the rapid decline in the amount of newly synthesized D1 protein. Previous studies revealed that the inhibition of PSII activity under strong light was due to an imbalance between the rate of photodamage to PSII protein and the rate of the repair of damaged PSII protein [52]. Excessive light exposure accelerated the generation of photodamaged D1 protein in the PSII reaction center, but inhibited the expression of chloroplastic FtsH proteases [43,52]. In this paper, the effect of exogenous ABA on *FtsHs* transcripts was markedly variable, depending on *FtsHs* isoforms and ABA concentration (Fig 11E–11K). Such diversity could play a coordinate/complementary role in regulating the degradation of the damaged D1 protein and in controlling the rate of D1 protein turnover during rice leaf senescence induced by varying ABA concentrations in senescent leaves. Interestingly, the response of *FtsH2* transcript to exogenous ABA incubation was similar to that of the *PsbA* transcript (Fig 11A and 11F), in addition to the similar changes in genotype-dependent temporal pattern during leaf senescence (Fig 10A and 10G). In *Arabidopsis*, FtsH2 protease, which is highly homologous with the protease encoded *by FtsH2* in rice based on the amino acid sequence and function structure [54], is the most abundant FtsH isozyme in chloroplasts, followed by *FtsH5* and *FtsH8*. By contrast, *FtsH1* is accumulated at only 10% of the *FtsH2* level [56]. The *Arabidopsis* mutant lacking either *AtFtsH2* or *AtFtsH5* could lead to leaf variegation, with *At*FtsH2 mutants being more variegated than *At*FtsH5 [21,53,55]. By contrast, the mutations in *AtFtsH1* and *AtFtsH8* did not cause any visual phenotype [53]. In this regard, we assume that *FtsH2* is likely to be one of the most important genes for the regulation of D1 protein turnover and PSII repair cycle in relation to ABA-induced leaf senescence, which may play a key role in the PSII repair cycle through the efficient degradation of photodamaged D1 protein.

## Supporting Information

S1 Fig Genotypic differences in O2- generation and H_2_O_2_ content and their temporal patterns in the flag leaves of the wild type and the *psf* mutant after anthesis. (A) O_2_^-^ generation. (B) H_2_O_2_ content, Error bars represent standard deviation (*n* = 3). * indicates significant difference (*P* < 0.05) between the wild type and the *psf* mutant.
(TIFF)

S1 Table Sequence of primer pairs used for real-time quantitative PCR in this study. (DOC)

S2 Table Effect of exogenous ABA treatment on pigment contents (Chl*a*, Chl *b*, Car) and Chl *a*/Chl *b* rate in the detached flag leaves of wild type and *psf* mutant, with distilled water incubation under illumination and darkness conditions as two controls. (DOC)
