# Supplementary figures and images for: Involvement of Abscisic Acid in PSII Photodamage and D1 Protein Turnover for Light-Induced Premature Senescence of Rice Flag Leaves
Source: PLoS One. 2016 Aug 17;11(8):e0161203. doi: 10.1371/journal.pone.0161203 (PMC4988704; doi:10.1371/journal.pone.0161203)

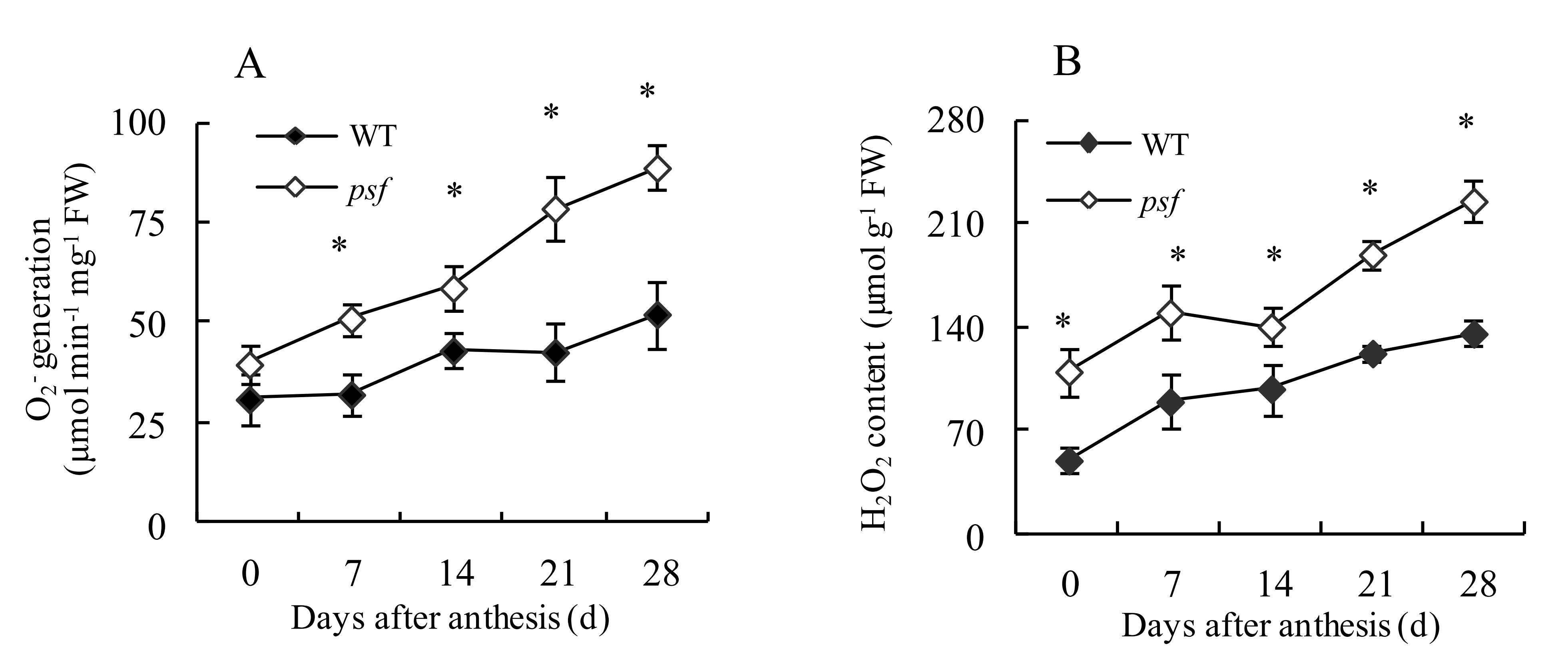

Supplement: S1 Fig — (A) O2- generation. (B) H2O2 content, Error bars represent standard deviation (n = 3). * indicates significant difference (P < 0.05) between the wild type and the psf mutant. (TIFF) [file pone.0161203.s001.tiff]
