# Supplementary material for: Involvement of Abscisic Acid in PSII Photodamage and D1 Protein Turnover for Light-Induced Premature Senescence of Rice Flag Leaves
Source: PLoS One. 2016 Aug 17;11(8):e0161203. doi: 10.1371/journal.pone.0161203 (PMC4988704; doi:10.1371/journal.pone.0161203)
Supplement: S1 Table — (DOC) [file pone.0161203.s002.doc]

**S1 Table. Sequence of primer pairs used for real-time quantitative PCR in this study.**

| Gene | Accession No. | Primer pairs | Products size (bp) |
| --- | --- | --- | --- |
| *Actin* | X16280 | 5'-CAGCACATTCCAGCAGATGT-3'  5'-TAGGCCGGTTGAAAACTTTG-3' | 198 |
| *PsbA* | DQ789329 | 5'-ATCTGTAGTTGATAGCCAAGGTCG-3'  5'-TAGGTCTAGAGGGAAGTTGTGAGC-3' | 118 |
| *PsbB* | GQ848589 | 5'-ACGGTGGAGTTCTATGGTGG-3'  5'-CCCTTGGACTGCTGCGAAA-3' | 154 |
| *PsbC* | X15901(9909-11330) | 5'-GGAGCAATGAACCTATTTGAAGTGG-3'  5'-GCCTAAGACTGCGGAGGAAAT-3' | 186 |
| *PsbD* | X15901(8900-9961) | 5'-AACCGCAGCAGTTTCCACC-3'  5'-CACCAACGAGTAAAATCCCCTT-3' | 93 |
| *OsFtsH1* | AK065019 | TCACATTCTTTGCTCCAAGC | 81 |
|  |  | CTACGGCCATTTGGTTCTCT |  |
| *OsFtsH2* | AK064913 | GCAGATGGTGGTTACATTCG | 104 |
|  |  | AGTTCCTTGCCATCATCCTC |  |
| *OsFtsH3* | AK100245 | GGTCACAATTGTTCCTCGTG | 143 |
|  |  | CAATCAAGACCTCCTCAGCA |  |
| *OsFtsH4* | AK072509 | GCAAGAGCAATGGTTACGAA | 123 |
|  |  | CTTCACCTCCTGCTCAATCA |  |
| *OsFtsH5* | AK120948 | AGCAAAGGCCTTGGTCTAAA | 129 |
|  |  | GGGATCTCGGAGGTAGTGAA |  |
| *OsFtsH7* | AK069509 | TACTCACGGAAATGGATGGA | 135 |
|  |  | CGGGAGCTTCAACCATAACT |  |
| *OsFtsH8* | AK069936 | ATGCTGCTTGAGAAGGAGGT | 125 |
|  |  | TCTTCTTCATCCTGGAACCC |  |

*PsbC* and *PsbD* were located in different sections of the chloroplast genome, respectively, in the range of 9909-11330 and 8900-9961.
