# Supplementary material for: Involvement of Abscisic Acid in PSII Photodamage and D1 Protein Turnover for Light-Induced Premature Senescence of Rice Flag Leaves
Source: PLoS One. 2016 Aug 17;11(8):e0161203. doi: 10.1371/journal.pone.0161203 (PMC4988704; doi:10.1371/journal.pone.0161203)
Supplement: S2 Table — (DOC) [file pone.0161203.s003.doc]

**S2 Table. Effect of exogenous ABA treatment on pigment contents (Chl*a*, Chl *b*, Car) and Chl *a*/Chl *b* rate in the detached flag leaves of wild type and *psf* mutant, with distilled water incubation under illumination and darkness conditions as two controls.**

|  | Time (d) | WT | | | *psf* | | |
| --- | --- | --- | --- | --- | --- | --- | --- |
|  |  | Dark | Light | ABA | Dark | Light | ABA |
| Chlorophyll *a* content (mg/g FW) | 0 | 2.90±0.07 |  |  | 2.86±0.05 |  |  |
|  | 3 | 2.80±0.17a | 1.42±0.30c | 2.33±0.21b | 1.73±c0.29a | 0.83±0.21c | 1.48±0.09b |
|  | 6 | 2.68±0.07a | 0.77±0.13c | 1.72±0.56b | 1.44±0.24a | 0.05±0.02c | 0.32±0.08b |
| Chlorophyll *b* content (mg/g FW) | 0 | 0.91±0.02 |  |  | 0.85±0.04 |  |  |
|  | 3 | 0.93±0.02a | 0.46±0.10c | 0.85±0.08b | 0.58±0.06a | 0.27±0.08c | 0.47±0.06b |
|  | 6 | 0.90±0.06a | 0.23±0.04c | 0.69±0.18b | 0.47±0.10a | 0.02±0.01c | 0.14±0.04b |
| Chl *a*/Chl *b* | 0 | 3.19±0.03 |  |  | 3.38±0.20 |  |  |
|  | 3 | 3.01±0.24a | 3.12±0.03a | 2.74±0.08b | 2.98±0.31b | 3.08±0.15a | 3.17±0.20a |
|  | 6 | 3.01±0.25b | 3.43±0.09a | 2.47±0.18c | 3.09±0.14a | 3.10±0.35a | 2.32±0.39b |
| Carotenoids content  (mg/g FW) | 0 | 0.53±0.01 |  |  | 0.52±0.02 |  |  |
|  | 3 | 0.54±0.02a | 0.37±0.05b | 0.48±0.07a | 0.36±0.01a | 0.27±0.09b | 0.39±0.01a |
|  | 6 | 0.54±0.02a | 0.20±0.02b | 0.49±0.03a | 0.32±0.03a | 0.06±0.01b | 0.27±0.03a |

Within a genotype, the different letters in the same row mean the signiﬁcant difference at *p* < 0.05.
